# Functional Feed for Laying Hens: Application of Saffron Extract as Eco‐Friendly Supplement With Cholesterol‐Lowering Properties

**DOI:** 10.1002/vms3.70155

**Published:** 2024-12-06

**Authors:** Reza Vakili, Amir Mokhtarpour, Seyed Ali Hosseini Ghaffari

**Affiliations:** ^1^ Department of Animal Science Kashmar Branch Islamic Azad University Kashmar Iran; ^2^ Special Domestic Animals Institute, Research Institute of Zabol Zabol Iran; ^3^ The Agricultural Faculty Agricultural Sciences and Resource Management in the Tropics and Subtropics (ARTS) University of Bonn Bonn Germany

**Keywords:** cholesterol, egg composition, gas emission, phenolic compounds, saffron petal extract

## Abstract

**Background:**

Saffron has been utilized in numerous studies as an additive to augment egg quality and to enhance the oxidative stability of egg yolk.However, there is limited knowledge on the responses of hens to dietary supplementation with saffron petals on egg chemical composition, fecal mineral excretion, and ammonia emission.

**Objectives:**

The objective of this study was to investigate the influence of saffron petal extract‐enriched diet on egg quality, blood metabolites and odorous gas emission from excreta in laying hens.

**Methods:**

The experimental methodology involved a feeding trial conducted over a period of 12 weeks, using 200 Hy‐line W36 laying hens aged 39 weeks. The dietary intervention included a basic diet (serving as a control with no supplementation), as well as diets fortified with 40, 60 or 80 parts per million (ppm) of hydroalcoholic saffron petal extract in a completely randomized design.

**Results:**

Results showed that the inclusion of saffron petal extract in the diet did not significantly affect the egg crude protein, fat and ash content. However, a significant reduction (*p* < 0.05) in yolk cholesterol concentration was observed. No substantial effect was noted on feed intake, feed conversion ratio (FCR) and egg weight (*p* > 0.05). On the other hand, a significant increase (*p* < 0.05) was documented in the egg production percentage of hens fed on the 80 ppm saffron petal extract diet compared to the control. Furthermore, saffron petal extract supplementation resulted in a significantly lower yolk cholesterol together with reduced serum cholesterol content (*p* < 0.05). Blood glucose and triglyceride concentrations also demonstrated a decrease subsequent to the inclusion of 80 ppm saffron petal extract. The excretion of faecal minerals did not show any significant alterations due to the dietary interventions (*p* > 0.05). Notably, hens supplemented with 60 and 80 ppm saffron petal extract displayed significantly diminished concentrations of faecal ammonia (NH_3_) emissions (*p* < 0.05) compared to the control.

**Conclusion:**

The study suggests that the inclusion of 80 ppm saffron petal extract in the diet of laying hens may serve as a functional food source to mitigate cholesterol levels in egg yolk and blood serum, as well as to reduce faecal ammonia emissions.

## Graphical abstract

• A total of 200 Hy‐line (W36) laying hens were assigned to four treatments, to investigate the influence of saffron petal extract‐enriched diet on egg quality, blood metabolites and odorous gas emission from excreta in laying hens.

• Saffron extract supplementation resulted in a significantly lower serum cholesterol content (*p* < 0.05).

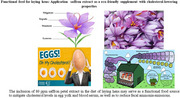

## 1 Introduction

Prior research has evidenced that the supplementation of herbal extracts may enhance laying function and egg quality (Dilawar et al. 2021). The surge in interest towards harnessing natural antioxidants to mitigate oxidative damage has been notable (Valko et al. 2007). Such therapeutic implications of antioxidant agents have stimulated scientific efforts to explore novel sources of natural antioxidants (Carocho and Ferreira 2013).

The perennial plant saffron (*Crocus sativus* L.), belonging to the family Iridaceae, is cultivated in several nations including Azerbaijan, China, France, Greece, Egypt, India, Iran, Israel, Italy, Mexico, Morocco, Spain and Turkey (Tuberoso et al. 2016). Its primary applications are as a food flavouring and colouring agent. Numerous studies have corroborated the antitumor, antiproliferative effects of saffron extracts on human cancer cells (Bhandari 2015; Vakili, Toroghian, and Torshizi 2022), in addition to their hypolipemic (Asdaq and Inamdar 2010) and hypoglycaemic activities (Elgazar, Rezq, and Bukhari 2013), largely attributable to key active constituents such as crocin, crocetin and safranal (Liakopoulou‐Kyriakides and Kyriakidis 2002). Saffron has been utilized in numerous studies as an additive to augment egg quality (Botsoglou et al. 2010) and to enhance the oxidative stability of egg yolk (Botsoglou, Florou‐Paneri, Nikolakakis et al. 2005; Goli, Mokhtari, and Rahimmalek 2012). A study conducted by Botsoglou, Florou‐Paneri, Botsoglou et al. (2005) found that, although saffron had no significant impact on feed intake and the feed conversion ratio (FCR) in laying hens, supplementation with 10 or 20 mg/kg of saffron mitigated lipid oxidation levels in eggshells.

The primary by‐product of the saffron processing industry, the saffron petal, is often discarded and remains unused (Kafi, Koocheki, and Rashed 2006). Owing to the presence of phenolic compounds such as crocin and kaempferol (Zeka et al. 2015), saffron petals exhibit antibacterial, antispasmodic, immunomodulatory, antitussive, antidepressant, antinociceptive, hepatoprotective, renoprotective, antihypertensive, antidiabetic and antioxidant activity (Goli, Mokhtari, and Rahimmalek 2012; Serrano‐Díaz et al. 2012; Hosseini, Razavi, and Hosseinzadeh 2018). When a hydroalcoholic extract of saffron petals was added to the diet of quail chickens, relative lymphoid organ mass, body weight and feed intake were enhanced, whereas ventricular fat and the FCR were reduced (Hosseini‐Vashan, Mohammadian, and Afzali 2018). Additionally, some studies report that supplementation with saffron petals resulted in decreased yolk cholesterol in laying hens (Jabbari Namroudi, Torki, and Mohammadi 2021).

Given the global saffron petal production of approximately 10,000 tons annually, and considering its relatively lower cost compared to saffron stigma (the primary commercial product of saffron), saffron petals present a suitable source for value‐added extraction applications in the health and food industries. Furthermore, the improvement of egg nutritional value via manipulation of poultry diets through the addition of natural antioxidant sources such as saffron petals may not only bolster animal and human health but also have a positive environmental impact. This is because the phenolic compounds in saffron petals can interact with gastrointestinal microbiome to mitigate ammonia emissions. However, there is limited knowledge on the responses of poultry to dietary supplementation with saffron petals. Therefore, the objective of this study was to assess the influence of diets containing different quantities of saffron petal extract on egg chemical composition, faecal mineral excretion and ammonia emission.

## 2 Materials and Methods

### 2.1 Extraction From Saffron Petals

In October 2021, saffron petals were gathered from the Torbat‐heydarieh district in the Khorasan Razavi province situated in north‐eastern Iran. Post‐harvest, the petals were shade‐dried and subsequently pulverized. The method employed for the extraction of saffron petals was elucidated in detail in our previous publication (Vakili, Toroghian, and Torshizi 2022). In brief, the pulverized petals were subjected to shaking with 50% aqueous ethanol in a 1:10 ratio for a period of 2 h. This mixture was then filtered and concentrated utilizing a rotary evaporator (Heidolph Laborota 4000, Schwabach, Germany). This was followed by spray‐drying, sieving and packaging processes. The yield of the ethanolic saffron petal extraction was 42%. The extract, in its powdered form, was stored in dark containers at 4°C until further use. Subsequently, the resultant powder was combined with calcium carbonate powder, in equal proportions, using a rapid mill composed of a ceramic jar with a lid and round ceramic balls at 350 rpm. Once the supplement was prepared, it was first mixed with a 10 kg diet in a small mixer, being remixed in a horizontal one with a capacity of 500 kg

### 2.2 Experimental Diets

UFFDA software was employed to formulate the diets on the basis of the requirements recommended for the strain (Table 1). The experiment spanned a duration of 12 weeks; the trial was initiated when hens were 39 weeks of age and terminated when they were 50 weeks old. Four experimental treatments were considered: basal diet + no saffron petal extract (control), and the control diet supplemented with 40, 60 and 80 ppm of saffron petal extract.

**TABLE 1:** Feed ingredients and chemical composition of basal diet (g/kg as fed).

Ingredients	g/kg
Corn	495
Soybean	280
Wheat	100
Calcium carbonate	85
Oil	8
Dicalcium phosphate	21
Sodium chloride	2.5
Sodium bicarbonate	1.5
Vitamin supplement^a^	2.5
Mineral supplement^b^	2.5
Methionine‐DL	2
Calculated and analysed chemical composition of diets (g/kg DM feed)	
Metabolizable energy (kcal/kg)	2696
Crude protein	172.4
Calcium	36.9
Available phosphorous	4.93
Sodium	1.8
Chlorine	1.8
Methionine	4.6
Methionine‐cysteine	7.44
Lysine	9.03
Threonine	6.5
Major bioactive compounds	
Total phenolic compounds (mg/g)	3.42
Total flavonoids (mg/g)	2.75
Kaempferol (% w/w)	12.6
Crocin (%w/w)	0.6
Anthocyanin (mg/L extract)	1712

^a^ In 1 kg of diet, vitamin supplement contained retinyl acetate: 3200,000 IU, cholecalciferol: 1320,000 IU, dl‐α‐tocopheryl acetate E: 8000 IU, Menadione: 1000 mg/kg, 3200 mg/kg calcium panthothenate, 12,000 mg/kg of niacin, 1000 mg/kg of vitamin B1, 2200 mg/kg of B2, 1600 mg/kg of B6, 360 mg/kg of B9, 9 mg/kg of B12, 30 mg/kg of biotin, 44,000 mg/kg of choline and 3000 mg/kg of antioxidant.

^b^ Mineral supplement contained 32,000 mg zinc, 80,000 mg manganese, 32,000 mg copper, 480 mg iodine, 88 mg selenium and 16,000 mg iron per kg basal diet.

### 2.3 Layers, Housing and Management

This study was carried out on the basis of procedures and guidelines approved by the Animal Care Committee of AREEO (Agricultural Research, Education and Extension Organization; No. IR. AREEO.14365.125.264). The experiment was implemented at research farm in Islamic Azad University Kashmar Branch, Iran. A total of 200 Hy‐line W36 layers, aged 39 weeks, were randomly allocated in three‐storey cages and subjected to four experimental treatments with five repetitions (10 hens per replication), using a completely randomized design (CRD). All hens were housed in wire cages measuring 52 × 34 × 30 cm on three floors, with ambient temperature around 20°C, 57% humidity and a lighting period of 16 h per day. Each cage was equipped with two nipple drinkers, and hens had unrestricted access to feed.

### 2.4 Measurement and Sampling

Throughout the experimental period, the average daily feed intake (g) and egg production (%) were recorded and utilized for the calculation of the FCR. According to the following formula, egg production was determined from 39 to 50 weeks on the basis of hen house as shown in the following formula:

$$\begin{array}{ccc} & & \mathrm{Hen}\;\mathrm{house}\;\mathrm{egg}\;\mathrm{production}\;\\ & & =\;\frac{\mathrm{Total}\;\mathrm{number}\;\mathrm{of}\;\mathrm{eggs}\;\mathrm{laid}\;\mathrm{during}\;\mathrm{the}\;\mathrm{period}}{\mathrm{Total}\;\mathrm{number}\;\mathrm{of}\;\mathrm{hens}\;\mathrm{housed}\;\mathrm{at}\;\mathrm{the}\;\mathrm{beginning}\;\mathrm{of}\;\mathrm{laying}\;\mathrm{period}} \end{array}$$

Egg weight was assessed using a digital analytical balance with a precision of 0.01 g. Every 4 weeks, 4 eggs from each replication were randomly collected to evaluate their proximate compositions, and results were the average values of combined measures obtained from each 4 week analysis. All eggs were manually broken, with half of them subsequently homogenized using a homogenizer (Ultra‐Turrax GMBH & Co. IKA Werke, Staufen, Germany), and the other half broken to separate the albumen and yolk.

During the final week of the experiment, fresh faecal droppings were collected in the morning from each cage using a sterile plastic bag, mixed, and then five representative samples were obtained for pH measurement. The remaining samples were stored at −20°C for subsequent laboratory analysis.

Approximately, 300 g of excreta was collected from each cage (five replicates per each treatment) in plastic zipper bags. These bags were placed in a plastic box equipped with a lid featuring two holes; one sealed with a membrane filter and the other used to measure ammonia. The samples were licensed to ferment at room temperature, and ammonia was recorded. This is from samples taken during the final week of the study and time zero is at the start of the ammonia emission assessment.

### 2.5 Laboratory Analysis

#### 2.5.1 Proximate Composition and Bioactive Compounds

The proximate composition of the eggs was analysed. The eggs were broken manually and then homogenized with a homogenizer. The contents of the egg were determined according to the Association of Official Analytical Chemists (AOAC 2005). The moisture (method 925.30), crude ash (method 900.02) and crude fat (method 925.32 using the Soxtec 2500, LabMakelaar Benelux B.V., Zevenhuizen, The Netherlands) of eggs, and also crude protein (method 925.31 using KjelMaster K‐375, BÜCHI Labortechnik AG., Flawil, Switzerland) of eggs, albumen and yolk were analysed, as per the AOAC (2005) guidelines.

The yolk cholesterol was determined enzymatically using a cholesterol reagent kit (Pars Azmon Cholesterol assay). The cholesterol content of the egg yolk was expressed as milligrams (mg) of cholesterol per egg.

The total anthocyanin content (TAC) was evaluated using the pH differential method outlined by Giusti and Wrolstad (2003). Briefly, 0.2 g of each sample was dissolved in 10 mL of distilled water in a volume flask far from the light. Absorbance was measured at 510 and 700 nm. The total phenolic content (TPC) of the extracts was determined by the Folin–Ciocalteau method (Singleton, Orthofer, and Lamuela‐Raventós 1999). TPC was presented as mg gallic acid (GA) equivalents/g. A calibration curve of GA (concentration range of 0.04–0.40 mg/mL) in methanol was prepared so that the TPC value could be obtained from the absorbance.

The total flavonoid content (TFC) was determined in accordance with the colourimetric assay of Spigno, Tramelli, and De Faveri (2007). Briefly, 0.5 mL of extracts was added into a 2.5 mL of water. Absorbance of the mixture was determined at 370 nm versus the prepared water blank. Quercetin was used as standard compound for the quantification of total flavonol. All values were declared as mg of quercetin equivalents per 100 g of sample.

#### 2.5.2 Faecal pH and Ammonia Gas Emission

For the determination of faecal pH, about 1 g of the faecal sample was diluted with 9 mL of distilled water, following which values were recorded using a portable digital pH‐meter (WinLab, Data Line pH‐meter; Windaus Labortechnik, Clausthal‐Zellerfeld, Germany). The ammonia (NH_3_) emission from excreta was measured according to the method proposed by Dilawar et al. (2021). In brief, faecal samples were allowed to ferment at room temperature, and a gas‐sampling pump (AP‐20, Gastec Corp., Kitagawa, Japan) equipped with a detector tube (3LA, 3 M) was employed to record ammonia emission at 0, 24, and 48 h. The NH_3_ concentration was expressed as ppm per 100 mL.

#### 2.5.3 Blood Metabolites

At the conclusion of the experiment, approximately 5 mL of blood was collected (sampled from the brachial wing vein after 4 h of starvation) in a glass tube (16 mm × 100 mm). Post‐clotting at room temperature, the serum was separated via centrifugation at 4000 × *g* for 3 min and kept at −20°C until further analysis. Serum concentrations of glucose, triglyceride, cholesterol, LDL and HDL were determined spectrophotometrically using commercial kits (Pars Azmon, Iran) with an auto‐analyser (Analyser A‐15 Biosystem, Barcelona, Spain).

### 2.6 Statistical Analysis

This experiment was conducted in a CRD and CRD with several observations in each repetition (proximate composition of egg) using the following models by the General Linear Model (GLM) procedure (SAS Institute Inc. 2001), respectively:

$$Y_{ij}=\mu +T_{i}+e_{ij}$$

$$Y_{ijk}=\mu +T_{i}+e_{ijk}+\varepsilon_{ijk}$$

where *μ* demonstrates mean trait, *T_i_* indicates the effect of treatment, and *ɛ_ijk_* and *e_ijk_* illustrate the effect of sampling and experimental error, respectively.

The collected data were subjected to statistical analysis using the General Linear Model (GLM) procedure of SAS (version 9.4; SAS Institute Inc. 2001). The treatment sums of squares in the ANOVA were further divided into their linear and quadratic effects. To compare the means of treatments, the Duncan multiple range test was employed whenever a significant *F*‐test was detected for means. The significance level was set at *p* ≤ 0.05, and trends were considered when 0.05 < *p* < 0.10.

## 3 Results and Discussion

### 3.1 Feed Intake and Laying Performance

In this study, the impact of saffron petal extract supplementation on feed intake and FCR was investigated. Although no statistically significant effects on feed intake and FCR were observed (*p* < 0.05), there was a noticeable decreasing linear trend (*p* < 0.1) with increasing saffron petal extract supplementation. Simply, the linear drop in feed intake could result in linear FCR improvement. Furthermore, saffron petal extract contained high levels phenolic compounds (3420 mg/kg) (Table 1) which may adversely affect feed intake as Biswas and Wakita (2001) reported that feed consumption was decreased by 15.9% in 1% green tea powder (containing phenolic compounds) fed broilers. This decreasing effect on feed intake following inclusion of 80 ppm saffron petal extract could be partly due to odour/taste effect of phenolic compounds in the extract. Yan, Meng, and Kim (2011) reported that strong odour caused a decrease in feed intake in swine when phenolic compounds were included at levels higher than about 1500 mg/kg diets. However, the mechanisms involved in this depression are not precisely understood. On the other hand, the bioactive components could decrease the activity of free radicals and break down fat, leading to an increase in the availability of nutrients, along with the growth of poultry (Windisch et al. 2008; Abdel‐Wareth and Lohakare 2014). These findings align with previous research in laying hens' diets which were also found to have no significant impact on feed intake and FCR (Bostoglou, Florou‐Paneri, Botsoglou et al. 2005; Jabbari Namroudi, Torki, and Mohammadi 2021). However, in broilers exposed to heat stress, the addition of saffron petal extract to the diet resulted in improved feed intake and FCR (Hosseini‐Vashan and Piray 2021;Hosseini, Naghous, and Hoseinyan Bilondi 2015). These discrepancies among studies may be attributed to variations in animal types and environmental conditions, such as temperature. Furthermore, the choice of extraction solvent (aqueous vs. alcoholic) can lead to different contents of phenolic compounds and flavonoids in saffron, subsequently affecting its antioxidant and antibacterial activities (Rahaiee et al. 2015).

Hens fed with 80 ppm saffron petal extract exhibited significantly higher egg production compared to those on the control diet (90.0° vs. 87.2%, *p* < 0.05). This increase in egg production may be attributed to the antimicrobial effects of kaempferol and crocin (Zeka et al. 2015), leading to improved gastrointestinal health in hens (Hosseini, Naghous, and Hoseinyan Bilondi 2015). However, contrasting results were reported by Bostoglou, Florou‐Paneri, Botsoglou et al. (2005) and Jabbari Namroudi, Torki, and Mohammadi (2021), who found no significant effect on egg production and egg weight in layers fed with saffron stigma or petal Table 2.

### 3.2 Proximate Composition of Egg

The chemical composition of egg is shown in Table 3. The amounts of protein in the egg, albumen and yolk were not significantly affected by the feeding treatments (*p* > 0.05). The moisture and ash content in the eggs were also not changed by the addition of saffron flower extract. However, a linear trend (*p* = 0.08) towards lower fat content in eggs with increasing dietary saffron extract was observed. The cholesterol concentration in egg yolk was decreased by supplementing the diet with petal saffron extract (*p* < 0.05) Table 3.

As far as we know, there are few data on the effects of saffron extracts on the chemical composition of eggs and cholesterol concentration in the yolk of laying hens. Consistent with the present results, Jabbari Namroudi, Torki, and Mohammadi (2021) reported that cholesterol content in yolk decreased in laying hens fed 1%, 2% and 3% saffron flowers. Dilawar et al. (2021) also found a decrease in cholesterol content in the yolk after adding increased amounts of *Mentha arvensis* and *Geranium thunbergii* extract to the drinking water of Hy‐Line Brown laying hens. In laying hens, cholesterol is synthesized in the liver and deposited in the egg after being released into the blood in the form of very low‐density lipoproteins (Kim et al. 2004). Therefore, all factors that affect the biosynthesis of cholesterol may also affect egg yolk content. An et al. (2018) reported that dietary supplementation with detoxified *Rhus verniciflua* juice (0.05%, 0.1% and 0.2%) significantly decreased yolk cholesterol in Hy‐Line Brown laying hens. However, oral administration of pravastatin, an inhibitor of 3‐hydroxy‐3‐methylglutaryl coenzyme A reductase (HMGR), decreased yolk cholesterol but not plasma cholesterol in ISA Brown laying hens (Kim et al. 2004). Similarly, Akbarian et al. (2011) reported the inhibitory effect of plant phenolic compounds on 3‐hydroxy‐3methylglutaryl coenzyme A, which is essential for cholesterol synthesis. Therefore, the observed reduction in cholesterol concentration in egg yolk as a result of saffron extract administration could be due to changes in cholesterol metabolism and/or its biosynthesis and secretion processes (Kim et al. 2004). It is important to note that the nutritional value and chemical composition of animal derivatives can be influenced by a constellation of factors including diet, genetic predisposition, age and management techniques (Yang et al. 2020). The results of this study suggest that the inclusion of saffron flower extract in the diet could lead to the production of eggs with lower cholesterol content, a trait that is highly sought after by health‐conscious consumers.

### 3.3 Plasma Blood Metabolites

Table 4 shows the effect of saffron flower extract on blood metabolites of laying hens. The concentrations of glucose, triglyceride, cholesterol and LDL were significantly decreased by the administration of saffron petal extract (*p* < 0.05). The antidiabetic and hypolipidemic activities of saffron may be associated with its active constituents such as phenolic compounds including crocin, flavonoids and kaempfrol (Table 2), as Razavi and Hosseinzadeh (2017) found that the unique properties of saffron in the treatment of metabolic syndrome are due to the presence of three major components (crocin, safranal and crocetin). Similar to our findings, saffron either as powder or extract was reported to significantly reduce blood glucose levels in laying hens (Jabbari Namroudi et al. 2021) and in both diabetic (Mohajeri, Mousavi, and Doustar 2009) and healthy rats (Arasteh et al. 2010). This hypoglycaemic effect of saffron can be interpreted through its role in glucose metabolism by activating AMP‐activated protein kinase/acetyl‐CoA carboxylase and also improving insulin sensitivity, thus stimulating glucose uptake in skeletal muscle cells (Kang et al. 2012). In addition, crocin and crocetin in saffron have been shown to scavenge reactive oxygen species, which may help pancreatic β‐cells to increase insulin secretion and reduce elevated blood glucose concentrations (Assimopoulou, Sinakos, and Papageorgiou 2005). In addition, the reduction of plasma and pancreatic TNF‐α and interleukin 1 beta concentrations after safranal administration (Hazman and Ovali 2015) in diabetic rats, together with the above‐mentioned factors, could explain the hypoglycaemic results obtained by adding saffron flower extract to the diet of laying hens. The well‐documented therapeutic application of saffron is its hypolipidemic properties (Rios, Recio, and Giner 1996). For example, Gainer and Jones (1975) demonstrated that serum cholesterol levels of rabbits fed a high‐fat diet were significantly reduced by intramuscular injection of crocetin. More recently, Hosseini‐Vashan and Piray (2021) found that the addition of saffron flower extract to the diet of broiler chickens decreased the plasma concentration of cholesterol, which is consistent with our results. Intraperitoneal injection of a hydromethanolic extract of saffron lowered serum cholesterol levels in male rats (Arasteh et al. 2010). In agreement with our results, Asdaq and Inamdar (2010) also reported that the concentrations of cholesterol and triglycerides decreased by oral administration of saffron or crocin in rats. It has been shown that crocin, as a competitive inhibitor, can selectively inhibit the activity of pancreatic lipase in rats with dietary hypertriglyceridemia and hypercholesterolaemia, thereby decreasing the absorption of fat and cholesterol (Sheng et al. 2006; Kadoglou et al. 2021). Crocin could also reduce cholesterol esters in macrophages and the uptake of oxidatively modified LDL, thus preventing the formation of foam cells that would promote the development and progression of atherosclerosis (Zhang et al. 2022). Bravo, Mañas, and Saura‐Calixto (1993) found that non‐extractable tannins may decrease cholesterol absorption and increase lipid excretion by impairing lipid digestion through the formation of complexes with fatty acids in male Wistar rats. In addition, the presence of phenolic compounds in saffron extract (Table 1) may inhibit pancreatic lipase and thus indirectly decrease triglyceride absorption.

**TABLE 2:** Effects of saffron petal hydroalcoholic extract (SPE) on feed intake and performance of layers.

Item	Feed intake (g/d)	Egg production (%)	Egg weight (g)	FCR
Treatment				
Control diet (CON)	116.3	87.2^b^	58.66	2.28
CON + 40 ppm SPE	108.0	86.6^b^	58.36	2.14
CON + 60 ppm SPE	108.7	84.6^b^	59.10	2.18
CON + 80 ppm SPE	106.6	90.0^a^	58.56	2.03
SEM	3.57	2.21	0.462	0.091
*p* value				
Treatment	0.26	0.0001	0.72	0.29
Linear	0.096	0.53	0.83	0.09
Quadratic	0.40	0.21	0.79	0.95

*Note*: A statistically significant difference is observed in the means with various letters (a, b) in each column (*p* < 0.05).

Abbreviations: FCR, feed conversion ratio; SEM, standard error of the means.

**TABLE 3:** Effects of saffron petal hydroalcoholic extract (SPE) on egg proximate composition (%) and cholesterol content (mg).

Item	Egg moisture (%)	Egg crude fat (%)	Egg crude ash (%)	Egg crude protein (%)	Albumen crude protein (%)	Yolk crude protein (%)	Yolk Cholesterol (mg/egg)
Treatment							
Control diet	76.4	5.10	0.98	12.36	28.54	11.32	221.08^a^
CON + 40 ppm SPE	76.6	5.01	0.94	12.25	28.5	11.43	186.4^b^
CON + 60 ppm SPE	76.7	4.85	0.97	12.42	28.26	11.10	169.8^bc^
CON + 80 ppm SPE	76.9	4.78	0.96	12.48	28.2	11.22	153.7^c^
SEM	0.31	0.109	0.021	0.147	0.179	0.065	8.28
*p* value							
Treatment	0.87	0.23	0.38	0.72	0.89	0.38	0.002
Linear	0.65	0.08	0.27	0.44	0.47	0.29	0.0003
Quadratic	0.98	0.92	0.30	0.58	0.98	0.96	0.28

*Note*: A statistically significant difference is observed in the means with various letters (a, b, c) in each column (*p* < 0.05).

Abbreviation: SEM, standard error of the means.

**TABLE 4:** Effects of saffron petal hydroalcoholic extract (SPE) on serum blood metabolites (mg/dL).

Item	Glucose (mg/dL)	Cholesterol (mg/dL)	Triglyceride (mg/dL)	HDL (mg/dL)	LDL (mg/dL)
Treatment					
Control diet	198.5^a^	148.4^a^	1243^a^	9.12	4.84^a^
CON + 40 ppm SPE	190.6^ab^	125.7^b^	1176^ab^	9.61	4.75^a^
CON + 60 ppm SPE	188.1^ab^	121.0^b^	1172^ab^	9.73	4.21^b^
CON + 80 ppm SPE	180.9^b^	112.3^b^	1131^b^	9.59	3.89^b^
SEM	2.39	4.17	15.26	0.139	0.125
*p* value					
Treatment	0.02	0.004	0.04	0.48	0.002
Linear	0.004	0.0009	0.008	0.26	0.003
Quadratic	0.82	0.20	0.57	0.30	0.41

*Note*: A statistically significant difference is observed in the means with various letters (a, b) in each column (*p* < 0.05).

Abbreviation: SEM, standard error of the means.

### 3.4 Faecal Mineral Excretion

Table 5 shows the effect of saffron petal extract on faecal mineral excretion of laying hens. Faecal mineral excretion was not affected by the treatments (*p* > 0.05). In contrast to our results, Cabuk et al. (2004) found lower faecal crude ash in broilers fed *Yucca schidigera* compared with the control group. It is well documented that polyphenolic compounds (tannins) can bind minerals, reducing their digestibility (McSweeney et al. 2001). However, few data are available on the effects of simple phenols on mineral binding capacity. In the current study, we found a numerical linear increase in zinc excretion (*p* < 0.1), which may be due to an interaction between minerals and phenols, because McDonald, Mila, and Scalbert (1996) reported that simple phenol compounds can bind to copper and zinc to form metal‐phenol complexes. Thus, despite some advantages of phenol‐metal complexes (e.g., antimicrobial properties and limitation of oxidation) (Alu'datt et al. 2018), they may be undesirable if they reduce the bioavailability of dietary minerals.

**TABLE 5:** Effects of saffron petal hydroalcoholic extract (SPE) on faecal mineral excretion (mg/g).

Item	Ash (%)	Ca (mg/g)	Fe (mg/g)	Mg (mg/g)	Zn (mg/g)	P (mg/g)
Treatment						
Control diet	21.09	331.3	13.08	6.507	1.106	33.44
CON + 40 SPE	20.87	338.7	11.81	7.852	1.190	36.83
CON + 60 SPE	18.15	331.23	11.15	10.313	1.437	44.07
CON + 80 SPE	22.13	310.76	12.15	8.562	1.369	38.34
SEM	0.691	5.922	0.687	0.815	0.0676	2.091
*p* value						
Treatment	0.17	0.40	0.82	0.45	0.29	0.31
Linear	0.94	0.21	0.60	0.26	0.097	0.22
Quadratic	0.11	0.26	0.45	0.36	0.57	0.26

*Note*: A statistically significant difference is not observed in the means with various letters‐ in each column (*p* < 0.05).

Abbreviation: SEM, standard error of the means.

### 3.5 Ammonia Gas Emission

In the present study, dietary supplementation with saffron petal extract significantly decreased NH_3_ production at all measured time points, as well as the linear decrease in dry matter content of manure (Table 6). In poultry houses, ammonia is mainly produced by the biodegradation of faecal uric acid (Atapattu, Lakmal, and Perera 2017). Various methods such as litter treatments, air scrubbers and neutralizers have been used to mitigate odour and gas emissions from litter, but these techniques are expensive. Saffron flower extract as an inexpensive feed additive was effective in reducing faecal ammonia emissions even at time zero. This could be due to the fact that the phenolic compounds from this by‐product alter the gut microbial ecosystem, leading to improved digestion and nutrient uptake, thus improving production performance. This is supported by our findings of higher egg production in laying hens fed 80 ppm saffron petal extract. Similarly, Dilawar et al. (2019), Dilawar et al. (2021) reported that the use of *M. arvensis* and *G. thunbergii* significantly reduced ammonia emission from the faeces of broilers and laying hens. Saeed et al. (2018) demonstrated that *Y. schidigera*, a plant rich in phenolic compounds, can reduce ammonia and faecal odour and pollution in poultry houses. Moreover, the lower ammonia emission in hens fed with 80 ppm saffron petal extract compared to the control group could be attributed to the numerically lower faecal pH (8.61 and 8.00), as pH plays an important role in ammonia emission (Saeed et al. 2018). The antimicrobial activities of phenolic compounds may also decrease the excreta pH and mitigate ammonia (Saeed et al. 2018). Bist et al. (2023) reviewed that lower pH and moisture content limit NH_3_ emission from poultry manure or litter which reduce the emission of NH_3_ into the atmosphere. Ammonia release from poultry farms is a concerning issue as it is a pollutant that can harm the well‐being of employees and animals, leading to decreased productivity (Dilawar et al. 2021).

**TABLE 6:** Effects of saffron petal hydroalcoholic extract (SPE) on faecal pH and dry matter (%) and ammonia emission (ppm) at 0, 24 and 48 h.

Item			Ammonia emission (ppm)^†^		
Treatment	Faecal pH	Faecal DM (%)	Hour_0	Hour_24	Hour_48
Control diet	8.61	27.9	149^a^	150^a^	165^a^
CON + 40 ppm SPE	8.31	27.8	89^b^	141^a^	133^b^
CON + 60 ppm SPE	8.09	24.8	90^b^	123^b^	131^b^
CON + 80 ppm SPE	8.00	21.6	78^c^	72.4^c^	70^c^
SEM	0.136	1.02	6.39	7.23	8.38
*p* value					
Treatment	0.42	0.09	<0.0001	<0.0001	<0.0001
Linear	0.11	0.02	<0.0001	<0.0001	<0.0001
Quadratic	0.71	0.39	<0.0001	<0.0001	<0.0001

*Note*: A statistically significant difference is observed in the means with various letters (a, b, c) in each column (*p* < 0.05).

Abbreviation: SEM, standard error of the means.

^†^ This is from samples taken during the final week of the study and time zero is at the start of the ammonia emission assessment.

## 4 Conclusion

Incorporating the hydro‐alcoholic extract of saffron petal at 80 ppm into the diet of laying hens elicited an enhancement in egg production and reduced serum cholesterol and yolk cholesterol, while maintaining a consistent feed intake that showed no significant deviations. In addition, serum glucose and triglyceride levels were decreased when saffron petal extract was included at 80 ppm. All doses of dietary saffron petal extract decreased the emission of harmful ammonia gas from excreta without significant effects on mineral excretion. Overall, it can be concluded that 80 ppm saffron petal extract can be added to the diet of laying hens as a functional food source.

## Author Contributions

**Reza Vakili**: study concept and design, drafting the manuscript, administrative, technical, and material support. **Amir Mokhtarpour**: acquisition of data, statistical analysis and interpretation of data. **Seyed Ali Hosseini Ghaffari**: review and editing.

## Ethics Statement

All research reported in this research has been conducted in an ethical and responsible manner and is in full compliance with all relevant codes of experimentation and legislation.

## Conflicts of Interest

The authors declare no conflicts of interest.

### Peer Review

The peer review history for this article is available at https://publons.com/publon/10.1002/vms3.70155.

## Data Availability

The data presented in this study are available on request from the corresponding author upon reasonable request.
